# Elucidating diversity in the class composition of the minicircle hypervariable region of *Trypanosoma cruzi*: New perspectives on typing and kDNA inheritance

**DOI:** 10.1371/journal.pntd.0007536

**Published:** 2019-06-27

**Authors:** Fanny Rusman, Nicolás Tomasini, Noelia-Floridia Yapur, Andrea F. Puebla, Paula G. Ragone, Patricio Diosque

**Affiliations:** 1 Unidad de Epidemiología Molecular (UEM), Instituto de Patología Experimental, Universidad Nacional de Salta-CONICET, Salta, Salta, Argentina; 2 Instituto de Biotecnología, Centro de Investigaciones en Ciencias Agronómicas y Veterinarias, Instituto Nacional de Tecnología Agropecuaria, Hurlingham, Buenos Aires, Argentina; Instituto de Investigaciones Biotecnológicas, ARGENTINA

## Abstract

**Background:**

*Trypanosoma cruzi*, the protozoan causative of Chagas disease, is classified into six main Discrete Typing Units (DTUs): TcI-TcVI. This parasite has around 10^5^ copies of the minicircle hypervariable region (mHVR) in their kinetoplastic DNA (kDNA). The genetic diversity of the mHVR is virtually unknown. However, cross-hybridization assays using mHVRs showed hybridization only between isolates belonging to the same genetic group. Nowadays there is no methodologic approach with a good sensibility, specificity and reproducibility for direct typing on biological samples. Due to its high copy number and apparently high diversity, mHVR becomes a good target for typing.

**Methodology/Principal findings:**

Around 22 million reads, obtained by amplicon sequencing of the mHVR, were analyzed for nine strains belonging to six *T*. *cruzi* DTUs. The number and diversity of mHVR clusters was variable among DTUs and even within a DTU. However, strains of the same DTU shared more mHVR clusters than strains of different DTUs and clustered together. In addition, hybrid DTUs (TcV and TcVI) shared similar percentages (1.9–3.4%) of mHVR clusters with their parentals (TcII and TcIII). Conversely, just 0.2% of clusters were shared between TcII and TcIII suggesting biparental inheritance of the kDNA in hybrids. Sequencing at low depth (20,000–40,000 reads) also revealed 95% of the mHVR clusters for each of the analyzed strains. Finally, the method revealed good correlation in cluster identity and abundance between different replications of the experiment (r = 0.999).

**Conclusions/Significance:**

Our work sheds light on the sequence diversity of mHVRs at intra and inter-DTU level. The mHVR amplicon sequencing workflow described here is a reproducible technique, that allows multiplexed analysis of hundreds of strains and results promissory for direct typing on biological samples in a future. In addition, such approach may help to gain knowledge on the mechanisms of the minicircle evolution and phylogenetic relationships among strains.

## Author summary

Chagas disease is an important public health problem in Latin America showing a wide diversity of clinical manifestations and epidemiological patterns. It is caused by the parasite *Trypanosoma cruzi*. This parasite is genetically diverse and classified into six main lineages. However, the relationship between intra-specific genetic diversity and clinical or epidemiological features is not clear, mainly because low sensitivity for direct typing on biological samples. For this reason, genetic markers with high copy number are required to achieve sensitivity. Here, we deep sequenced and analyzed a DNA region present in the large mitochondria of the parasite (named as mHVR, 10^5^ copies per parasite) from strains belonging to the six main lineages in order to analyze mHVR diversity and to evaluate its usefulness for typing. Despite the high sequence diversity, strains of the same lineage shared more sequences than strains of different lineages. Curiously, hybrid lineages shared mHVR sequences with both parents suggesting that mHVR (and DNA minicircles from the mitochondria) are inherited from both parentals. The mHVR amplicon sequencing workflow proposed here is reproducible and, potentially, it would be useful for typing hundreds of biological samples at time. It also provides a valuable approach to perform evolutionary and functional studies.

## Introduction

The protozoan parasite *Trypanosoma cruzi* (Kinetoplastea: Trypanosomatidae) is the causative agent of Chagas disease. This parasite infects millions of people throughout its distribution in Latin America. Chagas disease can display a broad pathological spectrum, including potentially fatal cardiological and gastrointestinal dysfunctions [1].

*T*. *cruzi* is a monophyletic taxon showing a remarkable genetic heterogeneity, with at least six phylogenetic lineages formally recognised as Discrete Typing Units (DTUs), TcI–TcVI [2, 3]; and a seventh lineage, named TcBat [4–6]. The genetic diversity of *T*. *cruzi* was firstly revealed by Multilocus Enzyme Electrophoresis [7, 8] and posteriorly by very diverse techniques including Multilocus Sequence Typing (MLST) [9–12], microsatellite typing (MLMT) [13–18], target-specific PCR [19–21], PCR-RFLP [22, 23], PCR-DNA blotting with hybridization assays [24–26], and recently by amplicon deep sequencing [27, 28]. The different approaches have their own advantages and disadvantages and bring out the genetic diversity of *T*. *cruzi* at different levels. Approaches that allow direct typing from biological samples (blood, tissues, etc.), avoiding parasite culture, are more suitable for clinical and epidemiological studies. However, nowadays there is no methodologic approach with a good sensibility, specificity and reproducibility for direct typing on biological samples.

Because there is usually a low number of parasites in infected tissues or blood samples, genetic markers with high number of copies are required to achieve good sensitivity of detection [29]. In this regard, *T*. *cruzi*, as all the kinetoplastids, has a unique and large mitochondrion which contains a complex network of DNA, the kinetoplastic DNA (kDNA). The kDNA represents approximately 20–25% of the total cellular DNA in *T*. *cruzi* and consists of two kind of circular DNA molecules: maxicircles and minicircles. Maxicircles contain mitochondrial genes characteristic of other eukaryotes [30]. Minicircles are present in tens of thousands of copies [31]. Each of them is organized into four highly conserved regions located 90° apart each other, and an equal number of hypervariable regions (mHVRs) interspersed between the conserved regions [32]. The highly conserved regions of minicircles have been widely used as targets for molecular detection of *T*. *cruzi* DNA. The used primers show a good sensitivity and specificity [29] and amplify a region of about 330 bp that totally include the mHVRs present between conserved regions. This amplified region has been used in hybridization assays (mHVR probes) and DTU-specific hybridization was observed only between isolates belonging to the same genetic group [25, 26, 33–35]. This specificity observed in hybridization assays suggests the presence of DTU specific sequences and even genotype-specific sequences (i.e. sequences showing specificity at intra-DTU level). However, technical limitations that existed until a few years ago for sequencing these highly variable kDNA regions, prevented the identification of the sequences in which the specificity relies. Some attempts were made by cloning and sequencing some mHVRs [36, 37] but the limited number of studied sequences were not enough to obtain a complete picture of the genetic diversity of these sequences. Thus, the observed hybridization patterns between mHVRs continue being a black box system and the sequence diversity of *T*. *cruzi* mHVRs virtually unknown.

Beyond the potential utility for strain typing, studying mHVR diversity is also interesting because these sequences are involved in functions that are only known in kinetoplastids and in no other eukaryotic organism. mHVRs code for short RNAs called guide RNAs (gRNAs). gRNAs are involved on edition of several mitochondrially-coded mRNAs. This edition varies from addition of some *Us* to building almost the full open reading frame of the mRNA [38, 39]. In this sense, gRNAs can be inferred from sequences of the mitochondrial mRNAs and diversity on edition among strains can be addressed [40]. In addition, studying mHVR diversity can shed light on how such sequences evolve and how they are inherited.

Here, we propose an amplicon deep sequencing approach that allows an accurate knowledge of the sequence diversity of the hypervariable region of kDNA minicircles of *T*. *cruzi* and opens the possibility of functional and evolutionary studies. This approach can be also used as a typing method for hundreds of samples at time.

## Materials and methods

### Strains

DNA from nine cloned *T*. *cruzi* strains belonging to the six main DTUs was examined in this study (Table 1). All the strains were typified by using an optimized Multilocus Sequence Typing scheme based on four gene fragments (*HMCOAR*, *GPI*, *TcMPX* and *RHO1*) according to Diosque et al. [7], in order to confirm DTU for each strain.

**Table 1:** Strains used in this study.

Strain	DTU	Origin	Host
**1. PalDa20cl3**	TcI	El Palmar, Argentina	*Didelphis albiventris*
**2. TEV55cl1**	TcI	Tres Estacas, Argentina	*Triatoma infestans*
**3. Esmeraldo**	TcII	Sao Felipe, Brazil	*Homo sapiens*
**4. TU18cl93**	TcII	Potosí, Bolivia	*Triatoma infestans*
**5. X109/2**	TcIII	Makthlawaiya, Paraguay	*Canis familiaris*
**6. CANIIIcl1**	TcIV	Belém, Brazil	*Homo sapiens*
**7. MNcl2**	TcV	Región IV, Chile	*Homo Sapiens*
**8. LL014R1**	TcV	Las Leonas, Argentina	*Triatoma infestans*
**9. LL015P68R0cl4**	TcVI	Las Leonas, Argentina	*Canis familiaris*

### Primer design and library construction

In order to amplify the minicircles hypervariable region, kDNA specific primers 121 (5’-ACACTCTTTCCCTACACGACGCTCTTCCGATCTAAATAATGTACGGG(T/G)GAGATGCATGA-3’) and 122 (5’-GTGACTGGAGTTCAGACGTGTGCTCTTCCGATCTGGTTCGATTGGGGTTGGTGTAATATA-3’) were modified by adding an oligo adapter to be used in an Illumina platform. The mHVR libraries were generated by a one-step PCR performed in 5 μl reaction volumes containing 5 ng of DNA, 250 nM of each primer, 2 μM of barcode primers, 5 U of Fast Start High Fidelity Enzyme Blend (Roche), 0.50 μl of 10X buffer (supplied with the Fast Start High Fidelity Enzyme Blend), 25 nM of MgCl_2_ (Roche), 0.25 μl of DMSO (Roche), 10 mM of PCR grade nucleotide mix (Roche). The PCR reaction was carried out on a Veriti Thermal Cycler (Life Technologies) and ran as follow: an initial denaturation step (10 min at 95°C), 10 cycles (95°C for 15 seconds, 60°C 30 seconds, 72°C 1 min), 2 cycles (95°C for 15 seconds, 80°C 30 seconds, 60°C 30 seconds, 72°C 1 min), 8 cycles (95°C for 15 seconds, 60°C 30 seconds, 72°C 1 min), 2 cycles (95°C for 15 seconds, 80°C 30 seconds, 60°C 30 seconds, 72°C 1 min), 8 cycles (95°C for 15 seconds, 60°C 30 seconds, 72°C 1 min) and 5 cycles (95°C for 15 seconds, 80°C 30 seconds, 60°C 30 seconds, 72°C 1 min). Amplicons were then purified using the magnetic beads Agencourt AMPure XP-PCR Purification (Beckman Genomics, USA). The concentration of the purified amplicons was controlled using Qubit Fluorometer 2.0 (Invitrogen, USA). All libraries were validated using the Fragment Analyzer system (Advanced Analytical Technologies, USA). The average size of the mHVR amplicons was ~480bp. All samples were then pooled and prepared according to the manufacturer's recommendations (Illumina Protocols: Sequencing Library Preparation) and sequenced on an Illumina MiSeq using a 500 cycle v2 kit (Illumina, San Diego, USA) to produce amplicons of approximately ~480 bp in length (250 bp paired-end reads).

### Bioinformatics

#### Read pre-processing

Reads were demultiplexed and adaptors were removed using Illumina Miseq Reporter, according to the manufacture recommendations. Raw sequence reads for all samples were quality filtered using the pair-end mode of Trimmomatic v0.36 [41]. This software was used to remove low quality bases from the beginning and end of sequence reads pairs (trimming). Also, a sliding window of 8 bases from left to right was performed. Sequence reads were cut whenever the average quality into the window fell below the threshold (<15, Phred score) and the right side of the read sequence was deleted. Sequences with a minimum read length of 150 nt, were retained. Then, the retained paired-reads were merged into a consensus sequence with its associated corrected base quality scores and chimeras were removed using LeeHom software [42] with default parameters.

#### Clustering

The following steps in the workflow were all performed using QIIME v1.9.1 [43]. A further quality check of sequence reads was carried with the “split_fastq_libraries.py” script. Default parameters were used, except for the quality threshold for trimming, which was raised to 25. Then, preprocessed sequences were clustered with the “pick_de_novo_otus.py” script. The *de novo* approach groups sequences based on sequence identity using the uclust algorithm [44]. Default parameters were used, and sequences were clustered according to three different identity thresholds—85%, 90% and 95%—in order to determine different mHVR clusters. The terms “minicircle class” or “mHVR class” are used in the bibliography without any clear definition and sometimes referring to mHVRs that codes the same gRNA. Here, we used the term “mHVR cluster” defined as a group of mHVRs that share a minimum sequence identity percentage with a cluster centroid without considering if a gRNA is coded by it. The mHVR cluster size is defined as the number of reads that belongs to such cluster. The following analyses were performed for these three identity thresholds.

Output tables were filtered at 0.005% using the “filter_otus_from_otu_table.py” script, in order to discard mHVR clusters with low abundance which are more probably sequencing artifacts [45], remaining parameters were used by default. The presence of a cluster into a strain was discarded when its abundance was lower than 20 read sequences. Diversity was measured by subsampling mHVR clusters tables using the “multiple_rarefactions.py” script. Clusters tables were rarefied including a maximum of 10,000 reads/sample in order to determine the minimum number of reads needed to detect all the clusters of mHVR. Alpha diversity measures—Simpson index and observed clusters—were estimated to determine the composition of each strain in sampling units using the “alpha_diversity.py" script. The output files of “alpha_diversity.py” were concatenated into a single file for generating rarefaction curves with the “collate_alpha.py” script followed by the “make_rarefaction_plots.py” script. In order to estimate compositional dissimilarity among strains, the “jackknifed_beta_diversity.py” script was used. Default parameters and the Bray-Curtis measure were chosen. The jackknifed beta diversity workflow calculates the beta diversity between each pair of previously resampled input strains, forming a distance matrix. The distance matrix then was visualized using UPGMA and Principal Coordinate Analysis (PCoA).

#### Rarefactions

In order to determine the minimum number of reads required to obtain the correct assignment of DTU for each strain, beta diversity was estimated at different subsampling levels. For each of the identity thresholds, the resampling of mHVR clusters was performed using decreasing amount of sequence reads, from 820,000 to 10,000 reads, with intervals of 10,000 reads and for 100 replications at each subsample.

#### Reproducibility assessment

To evaluate the reproducibility of the mHVR amplicon sequencing, two independent amplifications (PCR1 and PCR2) for LL015P68R0cl4 strain were performed. The data obtained were processed following the pipeline previously described. Clusters shared between PCR1 and PCR2 were evaluated, the Pearson’s correlation coefficient was calculated and the linear regression curve that best fitted the data was estimated.

## Accession numbers

The raw data set has been deposited in the NCBI SRA database (BioProject ID: PRJNA514922).

## Results

### mHVR abundance and diversity

A total of 22,092,382 paired reads were obtained by amplicon sequencing of the mHVR from nine strains belonging to six DTUs. A total of 14,766,753 sequences were retained (an average of ≈1.4 million of sequences per strain) after trimming low quality ends, merging paired reads (forward and reverse), elimination of chimeric reads and filtering by base quality (S1 Table). Surviving sequences were clustered according to different identity thresholds (85%, 90% and 95%) (Table 2, S2 and S3 Tables). The number of mHVR clusters for each strain was very similar using different thresholds (with differences less than 10% in all comparisons between 85% and 95% thresholds). However, clustering at 85% threshold returned few more mHVR clusters than clustering at 90% and 95% identity (See Table 2, S2 and S3 Tables). In addition, most clusters were highly divergent among them (S1 Fig). At any threshold, the number of mHVR clusters was variable among strains and DTUs (Table 2), ranging from 71 (Mncl2 –TcV) to 373 (X109/2 –TcIII) clusters. Additionally, strong intra-DTU variations in the number of clusters were observed in strains of TcI and TcII (Table 2). Finally, rarefactions of each dataset discarded that these differences among strains are the effect of different sequencing depths (Table 2, S2 and S3 Tables).

**Table 2:** Number of mHVR clusters defined at a threshold of 85% sequence identity for different strains.

	TcI		TcII		TcIII	TcIV	TcV		TcVI
	PalDa20 cl3	TEV55cl1	Esmeraldo	Tu18 cl93	X109/2	CANIIIcl1	LL014 R1	MNcl2	LL015 P68R0 cl4
mHVR clusters	324	234	347	151	373	149	72	71	108
Rarefaction at 820,000 sequences									
mHVR clusters	324	233.7	346.8	151	369	144.4	72	69.4	108
Simpson Index(diversity)*	0.991	0.989	0.994	0.978	0.994	0.885	0.827	0.902	0.942

* Average over 10 replications

**Fig 1:**
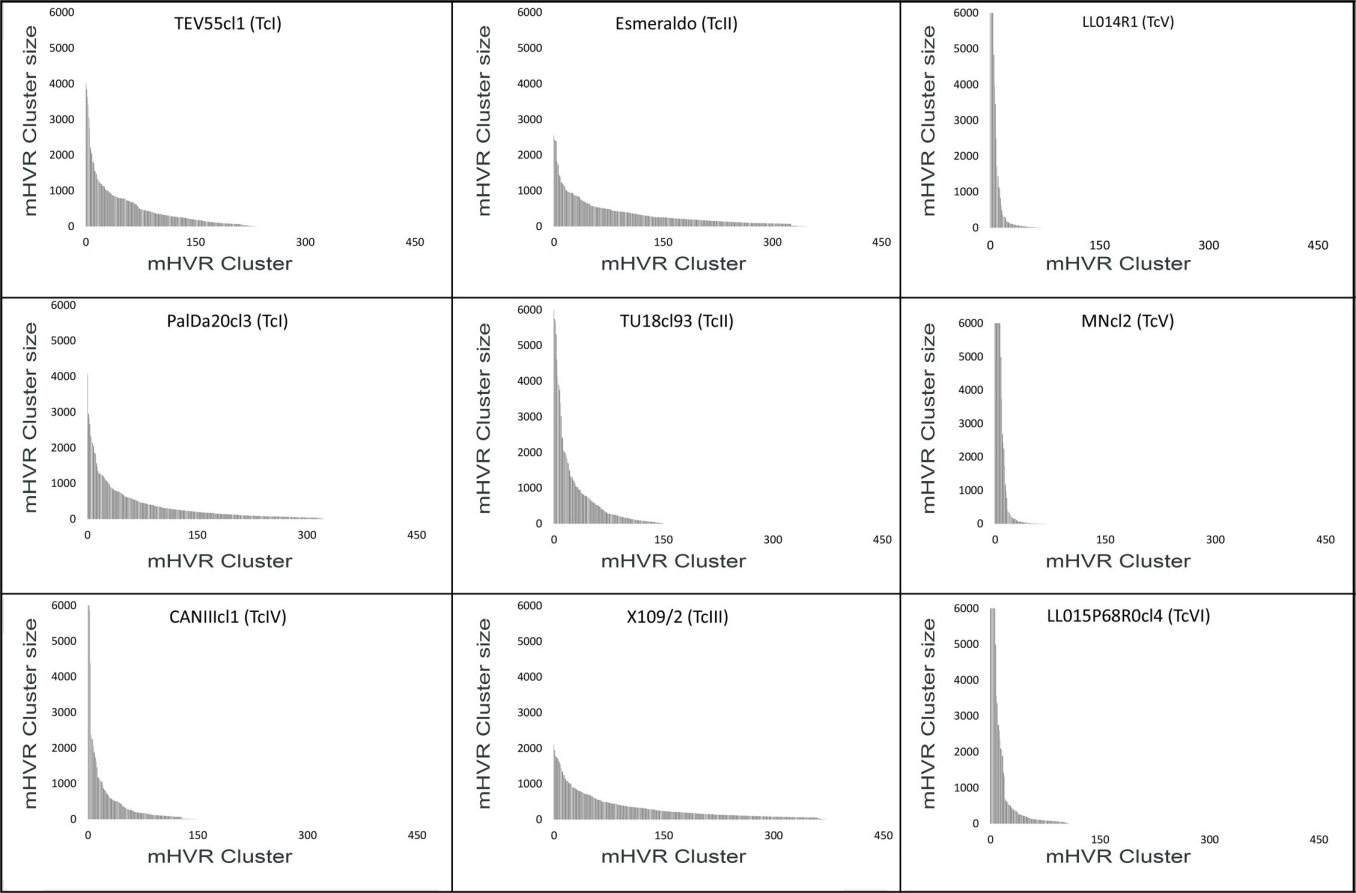
mHVR clusters distributed by size in nine strains. X-axis represent mHVR clusters ordered by decreasing size. The y-axis indicates the mHVR cluster size (i.e. number of reads in the cluster). The cluster size was standardized assuming a total of 120,000 mHVR sequences per parasite (i.e. the value represents the expected cluster size in a kDNA network with 120,000 mHVRs) in order to compare strains with different sequencing depths. Clusters with more than 6,000 sequences were observed for MNcl2, LL014R1, LL015P68R0cl4 and CANIIIcl1 but the bars were cut at this value in order to a clearer comparison among strains.

Strains belonging to TcIV, TcV and TcVI showed some dominant clusters containing a high proportion of reads (i.e. the cluster size) (Fig 1). The sum of the six most abundant clusters in TcIV, TcV and TcVI represent in all cases more than 50% of the clustered sequences (80.9% and 69.1% in the TcV strains LL014R1 and MNcl2, respectively; 58.7% in TcIV strain CANIIIcl1; and 52.5% in the TcVI strain LL015P68R0cl4). Even more, in LL014R1 and MNcl2 (TcV strains) the most abundant cluster represented the 29.7% and 17.8% of the total mHVR, respectively. Instead, none of the clusters present in TcI, TcII and TcIII strains represented more than 5.2%. This higher diversity in TcI, TcII and TcIII is also revealed by a higher Simpson diversity index than other DTUs (Table 2). Moreover, intra-DTU differences in mHVR cluster diversity were observed in TcII. Particularly, Tu18cl93 had relatively less cluster diversity than Esmeraldo (Table 2 and Fig 1).

### Shared and non-shared mHVR clusters at intra- and inter- DTU level

**Fig 2:**
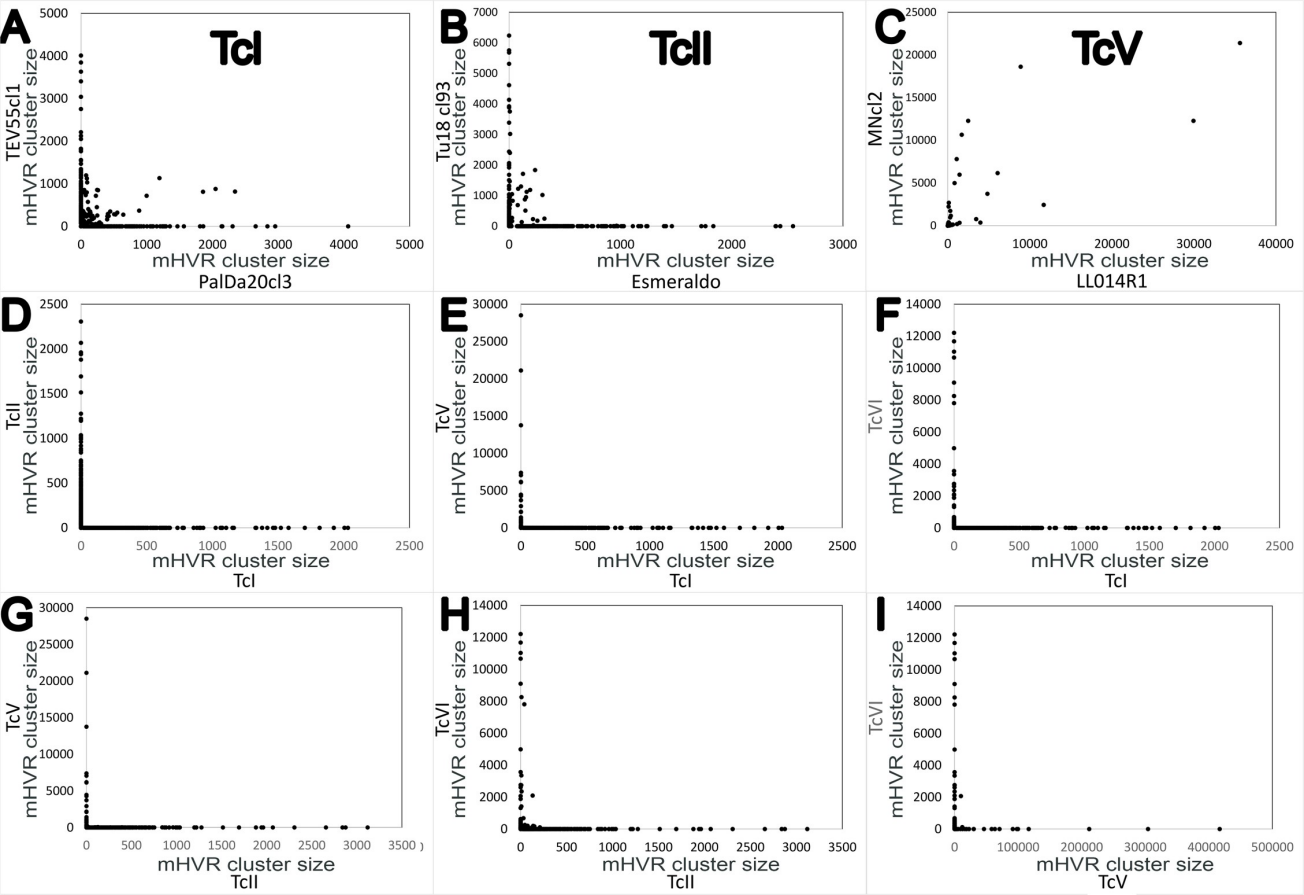
Strains belonging to the same DTU share more abundant mHVR clusters than strains of different DTUs (85% identity threshold). Each dot in the graph represents a mHVR cluster and the coordinates represent its standardized size in different strains (A-C) and in different DTUs of epidemiologic relevance (D-I). Dots that do not localize in the axes represent shared clusters. mHVR clusters for: TcI (A), TcII (B), TcV (C), TcI vs TcII (D), TcI vs TcV (E), TcI vs TcVI (F), TcII vs TcV (G), TcII vs TcVI (H) and TcV vs TcVI (I).

As expected, shared mHVR clusters were mostly observed in strains belonging to the same DTU. However, the percentage of shared clusters was highly variable depending on DTU. TcV strains (LL014R1 and MNcl2) showed the higher proportion of shared clusters (97.3%; 72/74). However, we observed strong differences in the cluster sizes (Fig 2C) although a positive correlation was detected (correlation coefficient, r = 0.75) and some shared clusters were highly abundant in both strains (Fig 2C). TcI strains (PalDa20cl3 and TEV55cl1) shared 17.5% (83/475), and TcII strains (Tu18cl93 and Esmeraldo) shared 7.1% (33/466). Conversely, when we look for shared mHVR clusters between strains belonging to different DTUs, we detected none or few shared clusters (Fig 2D–2I and S2 Fig).

### Strain clustering based on mHVR supports DTU-based classification and supports the hypothesis of biparental inheritance of minicircles in TcV and TcVI

**Fig 3:**
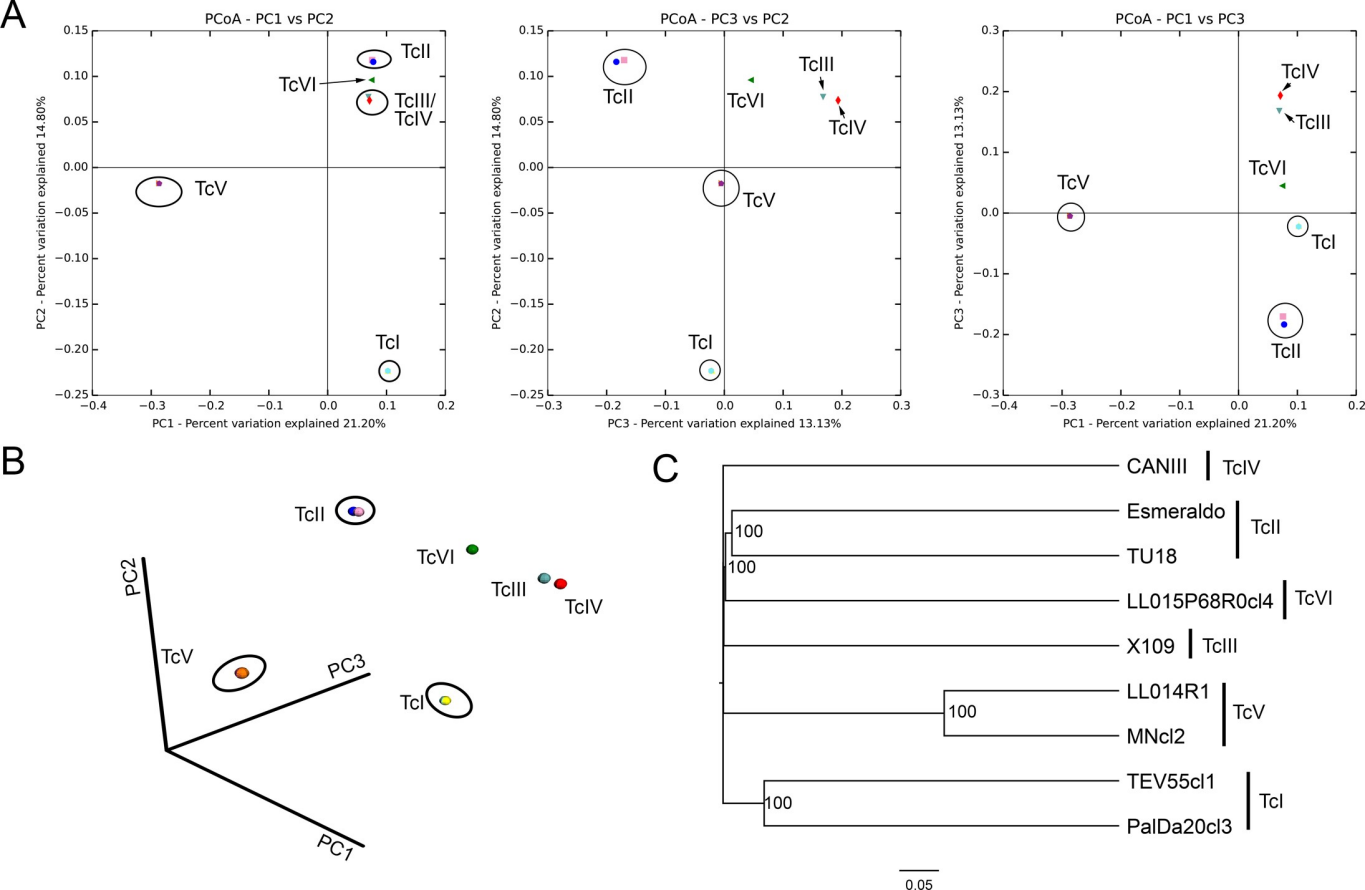
Principal coordinates analysis and UPGMA clustering. Both analyses were based on the mHVR clusters identified at a threshold of 85% for each strain. (A) 2D graphs combining two out of the three first axes resulting from PCoA. (B) Graph representing the three first axes of the PCoA. (C) Consensus UPGMA based on 10 rarefactions of the mHVR clusters at 820,000 sequences.

**Fig 4:**
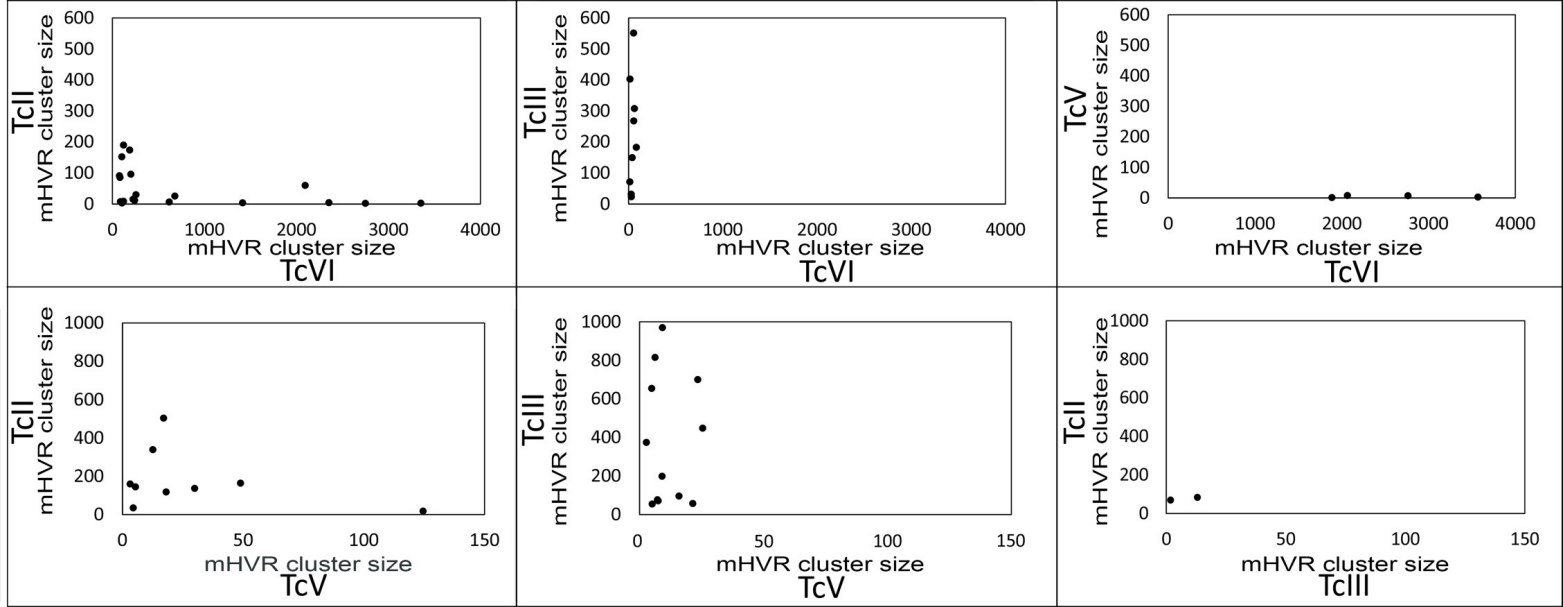
Shared mHVR clusters between parental (TcII and TcIII) and hybrids (TcV and TcVI) strains suggest biparental inheritance of minicircles in hybrids. Each dot represents a shared mHVR cluster (i.e. abundance > 0 in both analyzed DTUs). TcII, combination of Esmeraldo and Tu18cl93. TcIII, X109/2 strain. TcV, combination of clusters of LL014R1 and MNcl2. TcVI, LL015P68R0cl4 strain.

The Bray-Curtis dissimilarity between strains was calculated using mHVR clusters conformed at the different identity thresholds (85%, 90% and 95%). Such dissimilarities were used to analyze principal coordinates (PCoA) and to build UPGMA trees (Fig 3 and S3 Fig). Strains from the same DTU clustered together (Fig 3) despite the high dissimilarities between strains belonging to the same DTU (Fig 3C). These high dissimilarities between strains belonging to the same DTU determine that the three first axis in the PCoA explain just 49.1% of the variance. TcV strains clustered distant from other DTUs. TcIII and TcIV strains clustered near to each other. Interestingly, TcVI strain was placed between TcII and TcIII in the PCoA. Moreover, TcVI was clustered with TcII in the UPGMA tree (Fig 3C). Such results are not in agreement with the hypothesis of uniparental inheritance of the minicircles in the hybrid TcVI, which comes from hybridization between TcII and TcIII. Consequently, we analyzed shared clusters between TcII, TcIII and the hybrids DTUs (TcV and TcVI) in order to analyze the hypotheses of uniparental or biparental inheritance of minicircles. We used a 90% identity threshold in order to be more confident about the identity by descendance of the clusters. We observed that TcV and TcVI share 11/530 and 19/559 mHVR clusters with TcII, respectively. Likewise, TcV and TcVI shared 12/429 and 9/469 mHVR clusters with TcIII, respectively (Fig 4). Instead, TcII and TcIII share only 2 mHVR clusters between them out of a total number of clusters of 842 combining TcII and TcIII. These results suggest that minicircle inheritance is biparental. In addition, TcV and TcVI shared more mHVR clusters with their parental DTUs than between them (Fig 4) which is concordant with the hypothesis of independent origins of TcV and TcVI.

### Potential suitability of the amplicon sequencing for NGS-based typing of *T*. *cruzi*

**Fig 5:**
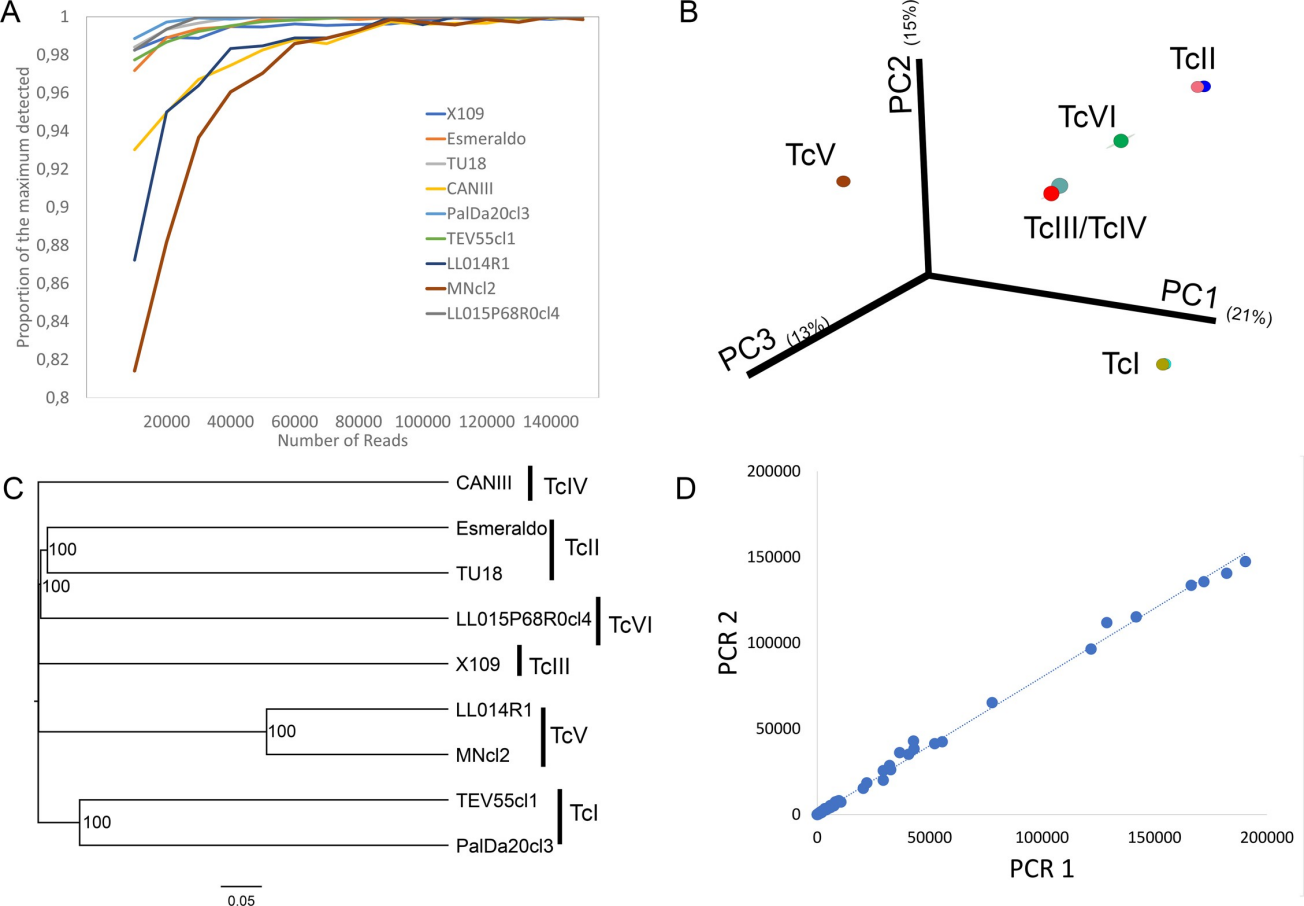
Suitability of amplicon sequencing of the mHVR for typing. (A) Proportion of clusters in relation to the maximum observed in Table 2 at different sequencing depths. (B) Principal Coordinates analysis based on Bray-Curtis dissimilarities showing the first three axes at a sequencing depth of 10,000 reads. (C) UPGMA based on Bray-Curtis distance showing relationships among strains at a sequencing depth of 10,000 reads. (D) Correlation between mHVR cluster sizes in two independent PCRs from the strain LL015P68R0cl4 (TcVI).

In order to test if parallel amplicon sequencing would be useful for simultaneous typing of hundreds of strains, we first evaluated rarefaction curves. In general, the minimum number of reads required to detect at least 95% of the observed clusters was 20,000 filtered reads. The only exception was MNcl2, which required 40,000 filtered reads. Increasing the number of reads per sample beyond 20,000 slightly increased the number of detected mHVR clusters (Fig 5A). In addition, we evaluated the minimum number of reads required to observe the right DTU assignment described in Fig 2. As few as 10,000 reads were enough to accurate clustering of the strains (Fig 5B and 5C) at 100% of the rarefactions.

Amplicon sequencing of the mHVR could be useful to identify intra-DTU clusters, particularly in TcV or TcVI where strains may have the same composition of mHVR clusters but with high differences in abundance of each one. In order to develop future methods to assign strains to intra-DTU clusters is pre-requisite that amplicon sequencing can be reproducible to determine mHVR cluster abundance. Consequently, we assessed reproducibility by sequencing and comparing two independent PCR reactions of the mHVR in LL015P68R0cl4 strain (TcVI). High correlation in cluster abundances in different PCRs of the same sample was observed (r = 0.999 for the three different identity thresholds) (Fig 5D).

## Discussion

Here, we made a deep amplicon sequencing of the hypervariable region of kDNA minicircles in the six main lineages (DTUs) of *T*. *cruzi*. To the best of our knowledge, this is the first time that these kDNA regions were sequenced at millions of reads of depth. Our results shed light on different and very interesting aspects of these intriguing DNA sequences. We accurately show the level of sequence diversity of mHVR within strains, between strains belonging to the same DTU, and between strains belonging to different DTUs. Although it was already known that mHVR were highly diverse [36], the magnitude of this diversity at the intra- and inter-DTU level has not been demonstrated with the high precision provided by an NGS approach, as we made here.

We propose a method for typing/elucidating intra-specific diversity of *T*. *cruzi* based on the deep sequencing of the hypervariable region of kDNA minicircles. The idea is based on the outdated but highly sensitive method of mHVR probes [25, 26, 35, 46–48]. Such probes are useful to detect *T*. *cruzi* diversity in biological samples. However, this methodology has the disadvantages of being technically cumbersome, relying on visual interpretation of bands and requiring representative strains of the diversity of *T*. *cruzi* in every assay (used as probes). The deep amplicon sequencing approach proposed here is reproducible and based on objective sequence data which can be stored in online databases. Also, the method is multiplexable for hundreds of samples at time and it would be directly applied to biological samples as the mHVR probes. The method may be potentially useful to address epidemiological questions about associations between intra-specific diversity and variability in clinical manifestations of the chronic disease or the different rates of congenital transmission in different endemic areas. Such questions have been unsuccessfully addressed using molecular markers with low resolution and/or low sensitivity on biological samples. We determined that around 20,000 filtered reads are enough to reveal most mHVR diversity in a strain and theoretically allowing for running hundreds of samples in a single run of a MiSeq with costs similar or lower than MLST. However, a wider set of strains belonging to the six main lineages must be studied. In addition, new bioinformatic methods of analysis will be required for a direct application of the method to biological samples.

In order to develop such typing method, we preliminarily analyzed and compared the diversity of mHVR sequences in reference strains of six DTUs and at millions of reads of sequencing depth. We observed that strains of the same DTU share more mHVR clusters than strains of different DTUs. However, unprecedented high differences in mHVR cluster composition was observed for strains of the same DTU with less than 20% of shared mHVR clusters in TcI and TcII. Instead, almost all mHVR clusters were shared between different TcV strains. In addition, the patterns of DTU specificity observed by using mHVR probes may be explained in TcV and TcVI by the presence of some shared and abundant clusters. Instead, considering the higher diversity and low abundance of clusters in TcI, TcII and TcIII, the global pattern of sequences is probably the responsible of specificity in the hybridization assays involving these DTUs.

Interestingly, our data revealed that diversity of mHVR sequences was variable even within a DTU. This was particularly evident in TcII, where the number of mHVR clusters in Esmeraldo strain doubled that of Tu18cl93. Such differences may be caused by long times in culture as it has been observed for other trypanosomatids [40, 49]. However, both strains were isolated in the eighties and although it is possible that they had different times in culture, such times would be not very different (i.e. not in the order of decades). According to this, we suppose that the observed difference in mHVR diversity between the two TcII strains is not due to long time in culture. In support of the hypothesis of no influence of the time in culture, we observed no differences in mHVR diversity between the two TcV strains examined, despite they have very different times of isolation and maintenance mode in the laboratory. One of them was isolated in the 1980s and subjected to long periods of maintenance in culture (Mncl2); and the other TcV strain (LL014R1) was isolated in 2008 and maintained in triatomine-mouse passages.

Our results also shed some light on the evolutionary mechanism determining the large genetic distances in mHVR sequences among strains and DTUs. The focus should be first placed on TcV strains which are identical according to MLST and which shared most mHVR clusters. Despite this, they strongly varied in relative frequencies of mHVR clusters. Such variations cannot be attributed to simple stochasticity of the PCR amplification because we observed good correlation between different PCR reactions from the same sample (Fig 5D). Consequently, it is probable that minicircle diversity is mainly driven by genetic drift. We propose that when two strains diverge, the frequencies of mHVR cluster varies stochastically, some clusters increasing their relative frequency and other decreasing it. The next step can be seen in strains of TcI which are more genetically distant than the TcV ones. Such TcI strains show clusters with high abundance in one strain and with very low (or null) abundance in the other one (look at most clusters located on the axes in Fig 2). Therefore, some clusters will be lost if such lost is not deleterious (i.e. replaced by a different mHVR class that codes a gRNA editing the same mRNA fragment). Thus, strains would diverge by variations in frequency of the mHVR classes faster than by changes in their sequences. These variations in the frequency of mHVR classes probably are not under selective pressure. mHVR frequency variations are apparently allowed because the effective edition of the mRNA is not dependent on the abundance of a minicircle [50, 51]. Variations in the frequency of mHVR classes have been also inferred for *T*. *brucei* and *Leishmania* [52] and by a theoretical study assuming random or partially random segregation of minicircles [53].

With the purpose of developing in the future DTU specific PCRs, we analyzed if different DTUs share common mHVR clusters. Telleria et al. [36] did not detected shared sequences between DTUs probably because the low sequencing depth. With a different approach, Velazquez et al. [37] detected that most abundant mHVR classes in CL-Brener (TcVI) were also present in other DTUs but in a considerably lower frequency. We detected shared mHVRs between different DTUs but we did not detect any sequence shared by the six DTUs. Interestingly, we observed shared clusters between TcVI and TcIII (2.1%). This is expected considering that TcIII is a parental DTU of the hybrid TcVI and maxicircle sequences of TcIII are closely related to the TcVI ones [54–58]. However, the TcVI strain also shared 2.5% of mHVR clusters with Esmeraldo strain (belonging to TcII, the other parental DTU of TcVI). Something similar is observed for the also hybrid DTU TcV (Fig 3). Instead, only 2 mHVR clusters were shared between TcII and TcIII strains (0.2%). This clearly suggests that although maxicircles have apparently uniparental inheritance in TcV and TcVI, minicircles were probably inherited from both parentals and some of them persisted for 60,000 years since hybridization [59]. Biparental inheritance of minicircles and maxicircles has been proposed for *Trypanosoma brucei* hybrids [60–62]. In this parasite, it has been observed that maxicircle and minicircle inheritance is biparental in hybrids. However, maxicircles (20–50 copies) are homogenized by genetic drift resulting in the loss of whole maxicircles of one parental in few generations. However, minicircles have much more copies and they resist the fixation effect of genetic drift for more time. Consequently, maxicircle inheritance is biparental and just seems to be uniparental due to genetic drift. As consequence of the biparental inheritance of minicircles, it has been proposed that such inheritance may help to preserve mHVR diversity in *T*. *brucei* preventing the effect of the drift, and even that *T*. *brucei* requires genetic exchange to prevent the deleterious effect of loss of essential minicircle classes [53]. Nevertheless, genetic exchange has remained elusive to be detected in *T*. *cruzi*. Experimental hybrids obtained by Gaunt and coworkers showed that maxicircles are from one parental but minicircles were not analyzed [63] and kDNA inheritance was still not addressed in more recent experimental hybrids [64]. In addition, the frequency of genetic exchange may be variable among different DTUs. TcV and TcVI (which display a clearly clonal genetic structure at population level) [9, 10, 12, 57] have very low mHVR diversity. Instead, TcI, TcII and TcIII, for which genetic exchange has been proposed in the nature [11, 13, 15, 65], have higher mHVR diversity.

Moreover, our data may help elucidate the origin of hybrid DTUs. It has been proposed that TcV and TcVI are the result of a single hybridization event between TcII and TcIII and both DTUs diverged posteriorly [66, 67]. However, the alternative hypothesis (two independent hybridization) gain weight in the last years. Particularly, Multilocus Microsatellite Typing (MLMT) and Multilocus Sequence Typing (MLST) analyses favored the two independent hybridizations hypothesis [57, 59]. Considering biparental inheritance, and assuming a single hybridization event, the two hybrid DTUs (TcV and TcVI) should share more mHVR classes between them than with the parentals. However, our analyses show the contrary with very few classes shared between TcV and TcVI (Fig 4). This result supports independent hybridizations for the origin of TcV and TcVI. Alternatively, because both DTUs would have lost many mHVR clusters, the high divergence among them may have been caused by simple stochasticity, although is less likely. Interestingly, if minicircle are biparentally inherited it is expected that they will behave like the nuclear genes. So, it is expected that nuclear phylogenies will be similar to the mHVR phylogeny and both discordant to maxicircle phylogeny in cases of hybridization or introgression. However, some hypotheses about events that occurred very distant in time (e.g. mitochondrial introgression in the origin of TcIII [57–58]) might not be addressed by mHVR-based phylogenies because the almost null number of shared mHVR clusters between some DTUs.

Concluding, massive amplicon sequencing of the mHVR is reproducible and suitable for typing hundreds of *T*. *cruzi* strains at time because few thousands of reads are required per sample. However, some drawbacks still need solution. The main problem in biological samples are mixed infections of different genotypes or DTUs which are very frequent [48]. However, such problem can be overpassed by developing new bioinformatic methods comparing mHVR composition of a sample against a reference mHVR database which should collect information about the diversity in the DTUs of *T*. *cruzi*. In addition, the develop of an online database where mHVR representative sequences are stored is needed. We are currently working on such items. In addition, some rare events of mitochondrial introgression observed in natural populations of *T*. *cruzi* lead to discordant typing between nuclear and maxicircle markers [16, 68, 69]. However, it is unknown the effect of mitochondrial introgression on minicircles. In this sense, a Multilocus deep Sequence Typing (MLdST) may be good alternative and a second step. The deep sequencing of amplicons of the mHVR plus satDNA (a 195 bp sequence with 10^5^ sequences per genome) [70] may help elucidate such rare events and may increase sensitivity for typing on biological samples.

## Supporting information

S1 Table Reads obtained after different steps in the pipeline. (PDF)

S2 Table Number of mHVR clusters defined at a threshold of 90% sequence identity for different strains. (PDF)

S3 Table Number of mHVR clusters defined at a threshold of 95% sequence identity for different strains. (PDF)

S1 Fig Neighbor-Joining tree showing genetic distance among different mHVR clusters defined at 85% identity threshold in strain MNcl2. One representative mHVR sequence from each cluster was selected and aligned using MEGA v7 with default parameters. Uncorrected p-distances were used, and gaps were ignored in pairwise comparisons. Value above branches indicates their length and values under branches indicates their support calculated by 100 bootstrap replications.
(TIFF)

S2 Fig Shared and non-shared mHVR clusters between TcIII, TcIV and other DTUs at 85% identity threshold. Dots that do not localize in the axes represent shared clusters. The axis scales are different among Figs and they were set according the mHVR cluster with higher number of sequences.
(JPG)

S3 Fig Principal coordinates analyses at 90% and 95% identity thresholds. (JPG)
