# Supplementary material for: Elucidating diversity in the class composition of the minicircle hypervariable region of Trypanosoma cruzi: New perspectives on typing and kDNA inheritance
Source: PLoS Negl Trop Dis. 2019 Jun 27;13(6):e0007536. doi: 10.1371/journal.pntd.0007536 (PMC6619836; doi:10.1371/journal.pntd.0007536)
Supplement: S1 Table — (PDF) [file pntd.0007536.s001.pdf]

Table S1. Reads obtained after different steps in the pipeline

| | TcI | | TcII | | TcIII | TcIV | TcV | | TcVI | |
|---|---|---|---|---|---|---|---|---|---|---|
| Strain | PalDa20cl3 | TEV55cl1 | Esmeraldo | Tu18cl93 | X109/2 | CANIIIcl1 | LL014R1 | MNcl2 | LL015P68R0cl4[2] | |
| | | | | | | | | | PCR 1 | PCR 2 |
| Paired Reads | 2,728,193 | 2,305,460 | 2,328,934 | 2,307,362 | 2,551,201 | 1,450,910 | 2,003,702 | 2,442,318 | 2,192,191 | 1,782,111 |
| Trimmed and merged | 2,545,361 | 1,366,928 | 1,338,561 | 2,143,482 | 1,651,245 | 1,364,041 | 996,499 | 1,161,607 | 2,052,980 | 1,672,075 |
| surviving reads after filtering | 2,356,494 | 1,202,355 | 1,201,724 | 2,000,770 | 1,492,887 | 1,267,374 | 827,756 | 982,028 | 1,895,845 | 1,539,520 |
| Clustered[1] | 2,338,845 | 1,176,820 | 1,130,408 | 1,989,294 | 1,442,654 | 1,211,349 | 821,081 | 972,706 | 1,871,580 | NA |

1. Number of sequences that were included into mHVR clusters defined at the 85% identity threshold.
2. Two independent PCR amplifications were made in order to determine reproducibility. Only reads from PCR 1 were used for comparisons with other strains.
