# Supplementary material for: Elucidating diversity in the class composition of the minicircle hypervariable region of Trypanosoma cruzi: New perspectives on typing and kDNA inheritance
Source: PLoS Negl Trop Dis. 2019 Jun 27;13(6):e0007536. doi: 10.1371/journal.pntd.0007536 (PMC6619836; doi:10.1371/journal.pntd.0007536)
Supplement: S2 Table — (PDF) [file pntd.0007536.s002.pdf]

Table S2. Number of mHVR clusters defined at a threshold of 90% sequence identity for different strains

	TcI		TcII		TcIII	TcIV	TcV		TcVI
	PalDa20 cl3	TEV55cl1	Esmeraldo	Tu18 cl93	X109/2	CANIIIcl1	LL014 R1	MNcl2	LL015 P68R0cl4
mHVR Clusters	320	227	347	151	371	142	69	67	107
Rarefaction at 820,000 sequences									
mHVR clusters	320	225.8	346	151	366.3	138	69	65	107
Simpson Index (diversity)	0.991	0.989	0.994	0.978	0.994	0.885	0.827	0.901	0.942
