# Supplementary material for: Elucidating diversity in the class composition of the minicircle hypervariable region of Trypanosoma cruzi: New perspectives on typing and kDNA inheritance
Source: PLoS Negl Trop Dis. 2019 Jun 27;13(6):e0007536. doi: 10.1371/journal.pntd.0007536 (PMC6619836; doi:10.1371/journal.pntd.0007536)
Supplement: S3 Table — (PDF) [file pntd.0007536.s003.pdf]

Table S3. Number of mHVR clusters defined at a threshold of 95% sequence identity for different strains

	TcI		TcII		TcIII	TcIV	TcV		TcVI
	PalDa20 cl3	TEV55cl1	Esmeraldo	Tu18 cl93	X109/2	CANIIIcl1	LL014 R1	MNcl2	LL015 P68R0cl4
mHVR clusters	317	222	332	148	369	141	68	66	107
Rarefaction at 790,000 sequences									
mHVR clusters	309	217	322.3	144	353.9	135.1	66	60.8	102
Simpson Index (diversity)	0.991	0.989	0.994	0.978	0.994	0.886	0.829	0.905	0.942
