# Supplementary figures and images for: Elucidating diversity in the class composition of the minicircle hypervariable region of Trypanosoma cruzi: New perspectives on typing and kDNA inheritance
Source: PLoS Negl Trop Dis. 2019 Jun 27;13(6):e0007536. doi: 10.1371/journal.pntd.0007536 (PMC6619836; doi:10.1371/journal.pntd.0007536)

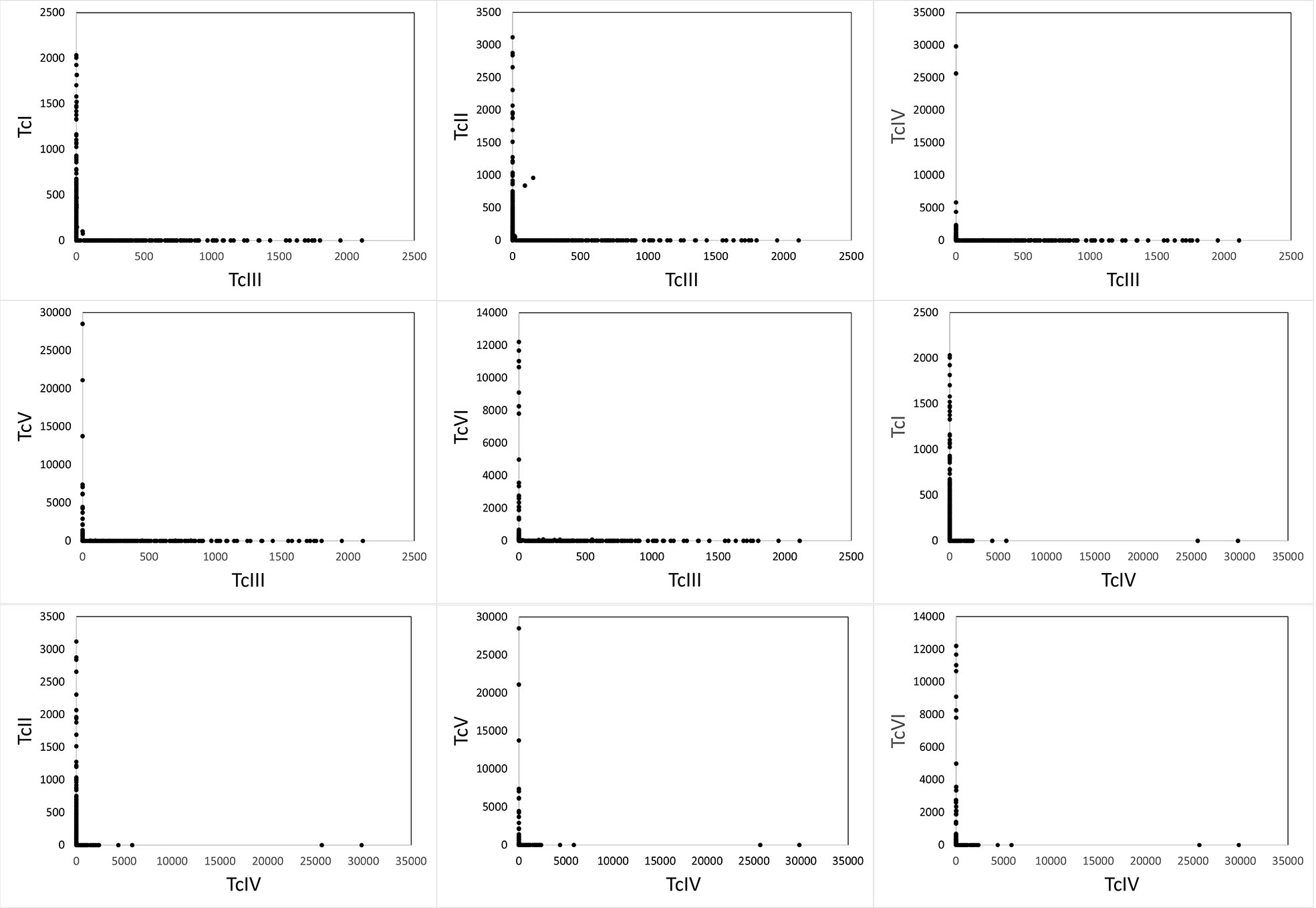

Supplement: S2 Fig — Dots that do not localize in the axes represent shared clusters. The axis scales are different among Figs and they were set according the mHVR cluster with higher number of sequences. (JPG) [file pntd.0007536.s005.jpg]
